# Children’s Mathematics and Verbal Self-concepts and Externalizing Behaviors: The Moderating Role of Peer Rejection at School

**DOI:** 10.3389/fpsyg.2017.01912

**Published:** 2017-11-01

**Authors:** Ylenia Passiatore, Teresa Grimaldi Capitello, Simona De Stasio, Michela Millioni, Simonetta Gentile, Caterina Fiorilli

**Affiliations:** ^1^Department of Human Sciences, Libera Università Maria SS. Assunta, Rome, Italy; ^2^Department of Neuroscience, Bambino Gesù Ospedale Pediatrico, Istituto di Ricovero e Cura a Carattere Scientifico, Rome, Italy; ^3^Department of Education, Roma Tre University, Rome, Italy

**Keywords:** math and verbal self-concept, peer rejection, behavioral problems, externalizing problems

## Abstract

Previous research has found a strong correlation between children’s academic self-concept and their behavioral problems. The present study examined whether children’s peer rejection moderated the relationship between children’s math and verbal self-concepts and their behavioral problems at school. We expected that children’s social competence, as measured by peer rejection, moderated the negative effect of low self-concept on children’s externalizing behaviors. Participants were 173 children (males = 93, *M*_age_ = 10.31 years, *SD* = 1.43). The main findings showed that peer rejection moderated the effect of both low verbal and math self-concepts on children’s externalizing behavior. The results are discussed in terms of the protective factor played by children’s social competence reducing the impact of low self-concept on children’s externalizing behaviors.

## Introduction

Existing literature has shown strong relationships between academic self-concept and students’ behavioral problems (e.g., Harter, 1993; Keiley et al., 2003; Marsh and O’Mara, 2008; Rockhill et al., 2009; Lee and Stone, 2012). The more children’s feel inadequate at school, the more likely they experience maladjustment behaviors. Although the domain-specific construct of academic self-concept is well-established in the research (e.g., Marsh et al., 2007) there is a lack of studies addressing whether low math and verbal self-concepts were differently related to behavioral problems. Still, not all children with low self-concept show behavioral problems. Interpersonal relationships at school and children’s rate of dislike among their classmates have been advanced as an important factor increasing the negative consequences of low self-concept (Zimmermann et al., 2017). The current study aimed to contribute to the literature by investigating whether children’s social relationships at school moderated the relationships between math and verbal self-concepts, and children’s externalizing behaviors.

### The Relations between Academic Self-concept and Behavioral Problems

Self-concept is a multidimensional construct that contains several representations of oneself originating from various social, cognitive, and affective experiences (Harter, 1993, 1999; Kaminski et al., 2005; De Stasio et al., 2014; Di Chiacchio et al., 2016). During childhood, self-concept is split into an academic self-concept and another self-concept involving social, emotional, and physical components (Marsh and Hattie, 1996; Camodeca et al., 2010).

Academic self-concept consists of three main domains: mathematic academic self-concept, verbal academic self-concept, and general academic self-concept (Marsh, 1990; Muijs, 1997), which involve a wide variety of different specific academic facets (e.g., math, biology, physical and economic sciences for math self-concept; writing/reading, text comprehension, foreign languages, history, and geography for verbal self-concept).

Previous studies have found that children’s negative academic self-concept increases the risk of behavioral problems affecting their quality of life beyond school-age (Harter, 1993; Marsh and O’Mara, 2008). Likewise, recent research has shown that low academic self-concept is strongly related to an increased risk to develop children’s social maladjustment (e.g., Lee and Stone, 2012), including low social competence and social support (Rockhill et al., 2009; Fiorilli et al., 2017), poor peer relations (Kiesner, 2002), and negative emotionality (Keiley et al., 2003). Overall, children who experience negative feelings about themselves may attempt to restore their a sense of self-concept through maladaptive forms of coping, such as aggression or delinquent behavior later in life (Marsh et al., 2001; Vermeiren, 2003; Donellan et al., 2005). More specifically, some authors (McGrath and Repetti, 2002; Ybrandt, 2008) have found that children’s negative academic self-perceptions were linked to subsequent internalizing/externalizing problems, with significant differences depending on age-related factors and gender differences. In fact, negative self-concept showed weaker effects in adults than in younger people (McGrath and Repetti, 2002; Lee and Stone, 2012). While, according to Ybrandt (2008) and Lee and Chung (2011), females and males with low self-concept are more likely to show internalizing and externalizing problems, respectively. Effectively, gender differences in children’s beliefs about mathematical competence beliefs are seen early in elementary school. For example, mathematical competence beliefs were stronger in boys than in girls, and this gender difference is unlikely to change over time. Therefore, even before they have much experience of different activities in more formal, evaluative settings such as school, boys, and girls have different competence beliefs. Moreover, gender-related, stereotypical beliefs concerning the distribution of mathematical talent (in favor of males) lead to a bias in adults’ perceptions of children’s competence, with effects on the children’s self-perception of their mathematical ability appearing as early as mid-elementary school years (Bandura, 1993; Tiedemann, 2000; Möller et al., 2009; Fiorilli et al., 2015, 2017; Di Chiacchio et al., 2016).

To sum up, existing literature highlighted the strong relationship between low self-concept in academic domains and increased risk of developing behavioral problems. Nevertheless to our knowledge there is a lack of investigation on the specific role played by both verbal and math self-concept on children’s adjustment at school. Effectively, while early research on children’s academic self-concept analyzed verbal-to-math as a continuum (Shavelson et al., 1976), recent empirical evidences lead to consider academic self-concept as domain-specific construct (see Marsh et al., 2007) with low inter-correlations each others (see Möller et al., 2009). Low self-concept in math as well as in verbal domains were related to children’s social relationships and reputation at school. These findings appear in line with early Marsh’s (1992) explanation about the process involved in shaping children’s academic self-concept in which a wide range of school experiences takes part, with a crucial role played by social relationships at school.

### The Role of Peers’ Rejection at School on Children’s Social Adjustment

School-age children spend a significant part of their lives at school where social interactions contribute to their adjustment (Rubin and Clark, 1983; Eisenberg et al., 1993). According to several authors children’s social status among their classmates plays a crucial role in their school-adjustment (e.g., Parker and Asher, 1987; Coie and Dodge, 1988). Effectively, being popular or well liked by peers is an indirect measure of children’s social competence and strongly related to their behavioral problems (Moreno, 1942; Denham et al., 1990; Rubin et al., 2011). Peer rejection is considered a negative experience that is detrimental to development (Parker and Asher, 1993; Parker and Gamm, 2003). Children who are rejected by many classmates exhibit high rates of internalizing and externalizing behavior (Coie et al., 1982; Cairns et al., 1988; Kupersmidt and Coie, 1990; Cillessen et al., 1992; Parkhurst and Asher, 1992; Dishion et al., 1995; Parker et al., 1995; Pettit et al., 1996; Miller-Johnson et al., 2002). Perhaps as a corollary of this, many children who are rejected by their peers become more isolated and less interactive over time, with the result that the peers then treat them more negatively and isolate them more often, perpetuating the risk of maladjustment (Buhs and Ladd, 2001).

Overall, peer’s acceptance may serve as a protective factor, which may reduce the impact of risk factors on adjustment outcomes (Henricsson and Rydell, 2006). Likewise, Stuhlman and Pianta (2009) claimed that classroom protective factors include peer relationships and the creation of a classroom community. Effectively, in several previous studies peer relationships have been found to moderate the effects of children’s problematic attitudes (e.g., anxiety, aggression, and difficult temperament) on their behavior problems (Miller-Johnson et al., 2002; Dodge and Pettit, 2003; Gazelle and Ladd, 2003; Ladd and Troop-Gordon, 2003; Henricsson and Rydell, 2006).

With regard to academic self-concept and peer acceptance at school, previous research has shown encouraging findings relating to the positive effect of peer acceptance on students’ self-concept. According to Gest et al.’s (2005) study peer academic reputations contributed to increase children’s academic self-concept suggesting that peer academic reputation play a role similar to those for teacher-rated academic skills as showed in previous literature (e.g., Harter, 1990; Skaalvik and Hagtvet, 1990; Hymel, 1997; Stanovich et al., 1998; Guay et al., 2003; Forrester et al., 2015). Recently, Zimmermann et al. (2017) have considered as relevant the role played by social dimensions in children’s academic self-concept development, assuming that social comparison relationships among classmates contributes to the development and adjustment of children’s academic self-concept. In other words, the risk factors of low self-academic concept may be ameliorated by classroom protective factors when children experience peer acceptance. Nevertheless, to our knowledge no information is available regarding whether peer relationships may be a risk or protective factor for children with low verbal and math self-concepts, respectively, on their behavioral outcomes.

### The Present Study

While the impact of the academic self-concept has been deeply analyzed in relation to students’ school-adjustment (Marsh et al., 2004; Donellan et al., 2005; Flook et al., 2005), it remains understudied how low children’s math and verbal self-concepts were, respectively, related to their behavioral problems. Furthermore, it is worth investigating whether social factors, as children’s social reputation at school, were able to moderate the negative impact of low self-concept on children’s behavior problems. The main purpose of the current study was to investigate whether children’s relationships with peers moderated the relationship between their academic self-concept (mathematics and verbal self-concepts) and externalizing behaviors. Based on the above-mentioned literature we assumed that children’s peer reputation at school played a moderating role between their low self-concept and externalizing behaviors. In the current study, the latter variable included all children’s behaviors related to problematic social interactions characterized by aggressiveness, deviance, and opposition, such as aggressive behavior, social problems, conduct problems, oppositional-defiant problems, and rule-breaking behavior. For the moderating variables, we focused on peer rejection among their classmates.

First, we expected that children’s verbal and math self-concepts, their peer rejection and externalizing behaviors were significantly associated. Children’s age and gender differences were also taken into account. Secondly, in accordance with our main purpose, using a path model, we tested whether children’s social competence moderated the relationships between the study variables. More specifically, we hypothesized that low peer rejection moderated the negative impact of low math and verbal self-concepts on their externalizing behaviors.

## Materials and Methods

### Participants

One hundred and seventy-three students from seven classes of a Primary and Middle School located in a middle-class urban area of central Italy participated in this study (93 males and 80 females). The average age of the participants was 10.31 years (*SD* = 1.43) ranging from age 9 to 12. All children were in their typical development stage with no diagnosed learning disability. Eighty-eight students were in the fourth year and 14 in the last year of Primary School. Seventy-one students were in the second year of the Middle School. The study was conducted in the ecological environment (at school) according to standard IRB protocol and did not cause any change of school routines.

### Instruments

#### Academic Self-concept

Self-concept in academic domains was measured using two sub-scales of the Self-Description Questionnaire-I (SDQ-I) Italian version (Camodeca et al., 2010). The sub-scales investigate children’s perception of their confidence and abilities in Mathematical and Italian language domains. Both sub-scales consist of 10 items on a Likert scale from 1 (false) to 5 (true) (e.g., “I’m interested in mathematics or in Italian”).

Cronbach’s alpha for math and verbal self-concepts for this sample were 0.92 and 0.91, respectively.

#### Peer Rejection

Peer rejection was measured by the Sociometry of Moreno (1951), an instrument created to highlight the social dynamics of class groups and the social relevance that each child has for the peer group. Each child indicated the names of three classmates that he/she would not invite to his/her party or would not involve in the class work-group. A score indicating the amount of ‘dislike’ choices given by their classmates was obtained for each child. A peer rejection index was obtained by standardizing the score per class (Coie et al., 1982).

#### Children’s Externalizing Behaviors

Teachers filled in the Child Behavior Check List 6/18 – Teacher Report Form (CBCL 6/18 – TRF) (Achenbach and Rescorla, 2001) for each child in every class. The scale consists of the following dimensions: rule-breaking behavior, aggressive behavior, social problems, conduct problems, and oppositional-defiant problems. Each sub-scale was measured on a three-point Likert scale (0 = absent, 1 = occurs sometimes, and 2 = occurs often). We calculated a total score of externalizing behaviors given by the sum of the average scores for each scale. Cronbach’s alpha for the total scale was 0.92.

### Procedures

The study began with the presentation of the research project to the Head of each school and to the teachers. The parents were informed about the aims of this study, since their approval was necessary in order to start the research. We sent permission forms home with the students and collected signed copies at the beginning of the research. Only students with active parental permission were allowed to participate in the study. Teacher participants provided signed informed consent.

### Analysis Strategy

Preliminary descriptive analysis and correlations were performed, using SPSS 23.0 program. Then we tested a hypothetical models through a path analysis, using M*plus* 7.00 (Muthén et al., 1998-2017) with Full Information Maximum Likelihood Estimation (FIML). In the model we investigated the moderating role of peer rejection in the relation between verbal and math self-concepts and externalizing behavior. In addition, to examine the moderation we included the interaction term between predictors and moderator (Aiken and West, 1991). To test the moderator hypothesis, it is important that the interaction term has a significant effect on the criteria, for the two predictors (predictor and moderator) the main effects are not relevant (Baron and Kenny, 1986). We considered model with CFI and TLI ≥ 0.95, RMSEA < 0.05 as good fitting model (Kline, 2010). In line with Cohen et al. (2003), all variables included in the model were mean-centered.

## Results

### Descriptive Statistics and Correlation Analysis

**Table 1** shows descriptive statistics and correlations among all study variables. As expected, significant associations emerged. Gender and age only present a relation with verbal self-concept. In particular, females and the younger participants seem to have a greater verbal self-concept than males and older participants. In **Table 1**, we can note that self-concepts in math and in verbal domains were positively correlated each other and negatively correlated with externalizing behaviors. In particular, children with high confidence in mathematical and verbal domains showed few externalizing behaviors. Nevertheless, no correlations were revealed between math and verbal self-concepts and peer rejection. Finally, peer rejection was highly and positively associated, as expected, to externalizing behaviors.

**Table 1:** Descriptive analysis and zero-order correlations.

	1	2	3	4	5	6
(1) Mathematic self-concept	1					
(2) Verbal self-concept	0.18^∗^	1				
(3) Externalizing behavior	-0.27^∗∗^	-0.17^∗^	1			
(4) Peer rejection	-0.12	-0.11	0.38^∗∗^	1		
(5) Gender	0.01	0.14^∗^	0.02	-0.06	1	
(6) Age	-0.05	-0.21^∗∗^	-0.11	-0.04	-0.08	1
Mean	3.81	3.76	56.19	0.96	1.46	10.31
SD	0.99	0.89	6.33	1.30	0.50	1.44

*^∗^*p* > 0.05; ^∗∗^*p* > 0.01.*

### Moderation Analysis

**FIGURE 1:**
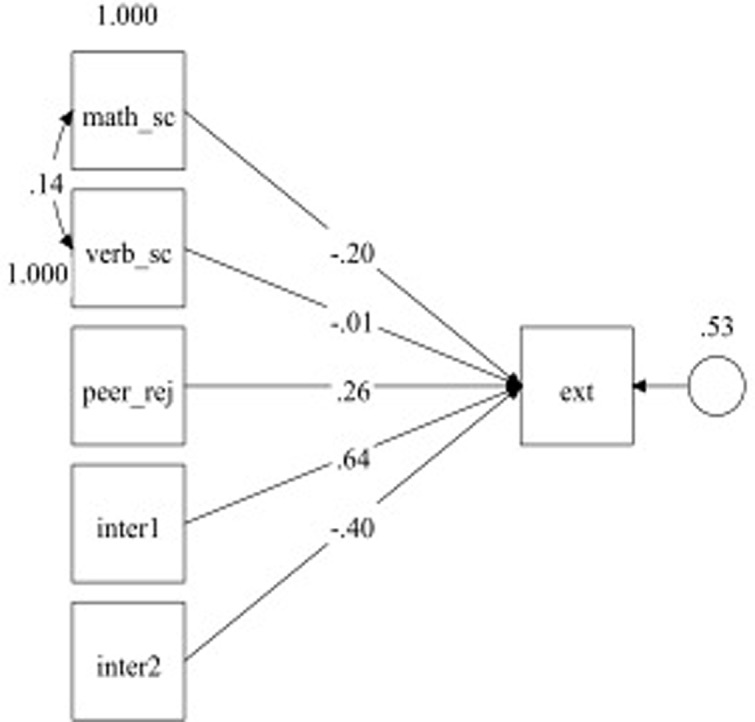
Path diagram of moderation analysis. Note: Math_sc, mathematical self-concept; Verb_sc, verbal self-concept; Peer_rej, peer rejection; Inter I, interaction between math_sc and peer_rej; inter2, interaction between verb_sc and peer_rej; Ext, externalizing behavior. All paths were significant with exception of the prediction from verb_sc on ext. *p* < 0.01.

In order to test the moderating hypothesis, a path analysis in M*plus* 7.00 was conducted using math self-concept and verbal self-concept as predictors, peer rejection as moderator, and children’s externalizing behaviors as criteria variable. Also the interaction terms were inserted as predictors. **Figure 1** shows the results of the model. Two children had missing values on math self-concept and on verbal self-concept, and for one child our dependent variable was missing. To account for these missing values and thereby retain these children in our analysis, we used multiple imputation in M*plus* 7.00 (Muthén and Muthén, 2006; see Enders, 2010).

In the Model the considerable amount of variance in externalizing behaviors was explained (*R*^2^ = 0.47, *p* < 0.001). Self-concept in math negatively predicted externalizing behaviors, whereas peer rejection positively predicted externalizing behaviors. Verbal self-concept didn’t predict our examined outcome. With regard to interaction terms, both were significant. Given the significance of interactions, we probed the effect of moderator on externalizing behaviors at low (-1 SD), medium, and high (+1 SD) levels of peer rejection (Cohen et al., 2003). Findings show that maths self-concept was negatively related to externalizing behaviors at low (*b* = -0.59, *p* < 0.001) and medium (*b* = -0.20, *p* < .001) levels but was positively related to externalizing behaviors at high (*b* = 0.25, *p* < 0.001) levels of peer rejection. At the same time, verbal self-concept was negatively related to externalizing behaviors at low (*b* = -0.50, *p* < 0.001) but not at medium (b = -0.01, *p* = 0.87) levels, and was positively related to externalizing behaviors at high (*b* = 0.83, *p* < 0.001) levels of peer rejection (see **Figure 1**). In sum, children with a greater perception of their mathematical and verbal skills showed minor externalizing behaviors, and this relation is especially true for children with lower rate of peer rejection within the class group. Nevertheless, if the child turns out to be highly rejected the relationship between the two variables changes with both low math and verbal self-concepts negatively associated to behavioral problems.

## Discussion

The main purpose of the current study was to investigate whether children’s peer reputation within their classmates group moderated the relationship between their academic self-concept (mathematics and verbal self-concepts, respectively), and their behavioral problems. First, we found positive correlations between math self-concept and verbal self-concept. Although some findings (Möller et al., 2009) considered math and verbal domains as separated and the age influence in their greater refining (Marsh et al., 1998), the scientific literature in this field is controversial and longitudinal researches could be useful to better understand the relation between the differentiation of the academic self-concept structure and the following development of the specific domains across age. Furthermore, our findings show the positive relation among age, gender, and verbal self-concept. Younger females present higher self-concept than older ones and males. Age and gender differences related to verbal domain are often frequent in favor of female (Skaalvik and Skaalvik, 2004) and younger students (e.g., Marsh et al., 1988). Also peer rejection and externalizing behaviors are positively related, while unexpectedly no correlations have been found between math and verbal self-concepts and peer rejection. In other words, children’s rate of peer rejection by their classmates was not associated with their low math and verbal self-concepts. Further researches are needed to investigate the direct relations between the children’s academic self-concept and their social relationships at school. It would be relevant understanding the separated role, the position and the weight of each factor (i.e., low academic self-concept, high peer rejection, and high behavioral problems) in determining the children general school adjustment.

Referring to our testing hypothesized model, we found significant findings which partially confirmed expected outcomes. When peer rejection has been analyzed as moderating variables significant associations emerged between low math and verbal self-concepts, respectively, and high risk of behavioral problems. In other words, low peer rejection could represent as protective factor in the relation between children’s low math and verbal self-concept and their externalizing behaviors. Our results may shed some light on the relation between children’s negative self-concept in mathematics and verbal domains and their risk of expressing externalizing problems taking into account the role of children’s social relationships as a relevant piece in their scholastic adaptation puzzle. Main results are discussed in more details below.

### The Protective Factors of Low Peer Rejection on Externalizing Behaviors

Children’s peer rejection moderated the relationship between children’s math and verbal self-concepts and their behavioral problems at school. Specifically, a low self-concept in mathematical or verbal domains is positively associated to aggressive, antisocial, and oppositional behaviors when children are highly rejected by their peers. Peer rejection is considered a stressful experience that can lead to negative outcomes due to feelings of isolation and failure to bond with conventional social institutions (e.g., Dodge and Pettit, 2003; Prinstein and Aikins, 2004). Children who are rejected by their peers may take more distance from the norms of conventional social institutions (such as school), putting them at risk for problem behaviors (Coie, 1990; Hinshaw, 1992; Masten et al., 2005). Conversely, a good social reputation among their peers could prevent children’s behavioral problems associated to a low self-concept in math and verbal domains. Children’s positive peer relationships improve the development of social skills, which can enhance academic self-concept (Raver, 2002; Sebanc, 2003). Good social interactions with peer can aid in the development of a set of social skills that will improve the likelihood for school success. These skills include conflict resolution, problem solving, and stress management skills (McClelland et al., 2000; Milteer et al., 2012). Development of these relationships and skills can be crucial for a child’s success or failure. Sebanc (2003) reports that good social skills can represent foundations for processing stress, solving problems, dealing with conflict that can help children regulate their emotions, engage in peer relationships, cope with difficult situations, and succeed academically. Since children who felt less confident about their academic competences may experience higher stress than their classmates with higher academic self-concept, learning how to deal with the possible stress, and developing strategies and skills to do so is crucial (Brooks-Gunn and Duncan, 1997; Duncan et al., 1998). Peer acceptance allows children to face greater chances for school success and to better cope with situations they encounter. If children can start to process stressful emotions, it may lower their behavioral disturbances as the classroom can represent a place of expression rather than repression.

Overall on the basis of the current study’s evidences we can assume that peers’ acceptance could represent a protective factor in the relation between low academic self-concept and high behavioral problems. This finding was partially consistent with previous research focusing on academic self-concept which predicted children’s behavioral problems with their classmates (Coie et al., 1982; Kupersmidt and Coie, 1990; Taylor et al., 2007; Fite et al., 2012, 2013).

In view of these findings, further analysis about the role of affective components in the relationship between the internalization of cognitive abilities, such as those required for verbal and mathematical competence, and the social skills related to adaptation in the school context would be interesting and useful to better understanding the complexity of the analyzed picture.

## Limitations and Future Implications

This study has some limitations. First, we have not considered the Italian and Mathematics grades given to the student by the teacher which may impact on influence children’s self-concept. Moreover, the grades matching the self-concept allow us to understand an under- or over-estimation of the child’s academic self-concept and to better understand its predictive role concerning maladaptive behaviors in class. Second, we have analyzed behavioral problems, such as rule-breaking behavior, aggressive behavior, social problems, conduct problems, and oppositional-defiant problems, which are externalizing behaviors, but have not considered internalizing ones. Both of these dysfunction domains were associated with impaired academic and social development in children (poor outcomes, peer relationship difficulties, underachievement, and poor personal adjustment) (Hinshaw, 1992; Mash and Barkley, 1996). As a future research direction, our findings supported one of the major principles of developmentally appropriate approaches, namely that all development domains (cognitive/academic, socio-emotional, and physical) are interrelated. Specifically, strengths and weaknesses of individuals, like academic self-concept, cannot be understood outside the social context in which their outcomes, like behavior problems, will be displayed. However, further research is needed to analyze in greater depth the mechanisms governing these relationships. Specifically, longitudinal studies focusing on causal and interrelated mechanisms may offer results that could be used to improve childhood education practices. Based on the results of our study, we believe that intervention programs designed to improve children’s schooling should not be limited to cognitive skills but should also involve social-emotional competences. According to several authors (for a review see Véronneau et al., 2014) children who live positive experiences at school have a higher chance of building confidence in their own abilities and to form the self-image of a good learner. This is especially true in these academic domains: mathematics and verbal abilities, in fact, represents both a cognitive and a social “visiting card,” starting in early childhood, for the growth of children’s social skills. Early and specific interventions could help prevent the adverse development of students’ behavioral problems, particularly aggressive, oppositional, and antisocial behavior.

## Ethics Statement

This study was carried out in accordance with the recommendations of the Declaration of Helsinki. Child assent and parental consent was obtained for all participating children. All adult participants gave written informed consent.

## Author Contributions

CF, YP, and SD designed the study, interpreted the results and wrote up the first draft of the manuscript. YP, MM, and TG analyzed the data, interpreted the results, and assisted in writing up the draft. CF and SG supervised the research and helped to interpret the data. All authors approved the final version of the manuscript.

## Conflict of Interest Statement

The authors declare that the research was conducted in the absence of any commercial or financial relationships that could be construed as a potential conflict of interest.
